# Case report of a patient with progressive multifocal leukoencephalopathy under treatment with dimethyl fumarate

**DOI:** 10.1186/s12883-015-0363-8

**Published:** 2015-07-08

**Authors:** Nele Dammeier, Victoria Schubert, Till-Karsten Hauser, Antje Bornemann, Felix Bischof

**Affiliations:** University Tübingen, Center of Neurology and Hertie Institute for Clinical Brain Research, Hoppe-Seyler Strasse 3, 72076 Tübingen, Germany; University Tübingen, Diagnostic and interventional Neuroradiology, Hoppe-Seyler Strasse 3, 72076 Tübingen, Germany; Institute for pathology und neuropathology, section neuropathology, Hoppe-Seyler Strasse 3, 72076 Tübingen, Germany

**Keywords:** PML, dimethyl fumarate, Psoriasis treatment

## Abstract

**Background:**

Progressive multifocal leukoencephalopathy is a severe demyelinating disease caused by the polyoma JC virus in patients with reduced immunocompetence. A few cases of progressive multifocal leukoencephalopathy have been reported in patients treated with fumaric acid esters.

**Case presentation:**

A 53-year-old Caucasian woman reported to our clinic with a first focal epileptic seizure and mild cognitive impairment. Since 1.5 years, she was treated with fumaderm for her psoriasis. During that time, her lymphocyte counts ranged between 450 and 700/μl. Cerebral magnet resonance imaging showed multifocal subcortical T2 hyperintense lesions with partial gadolinium enhancement. She did not have antibodies against human immunodeficiency virus 1 and 2 and cerebrospinal fluid-polymerase chain reaction for viral infections including a sensitive JC-virus polymerase chain reaction were negative. The diagnosis of progressive multifocal leukoencephalopathy was established by histological analysis and detection of JC-virus desoxyribonucleic acid in brain biopsy specimens. Dimethyl fumarate was stopped and Mirtazapin and Mefloquin were initiated. Neurological examination and imaging remained stable.

**Conclusions:**

Progressive multifocal leukoencephalopathy can occur in patients with lymphocyte counts between 450 and 700/μl, produce only faint symptoms and is not excluded by negative JC-virus-polymerase chain reaction in cerebrospinal fluid. The incidence of progressive multifocal leukoencephalopathy may thus be underestimated and a more careful surveillance of patients would be necessary.

## Background

Progressive multifocal leukoencephalopathy (PML) is a severe demyelinating disease of the central nervous system (CNS) caused by the polyoma JC virus in patients with reduced immunocompetence within the CNS. PML may develop in patients treated with immunomodulatory treatment such as natalizumab, rituximab, efalizumab, infliximab or mycophenolat mofetil [1]. Recently, a few cases of PML in patients with psoriasis treated with fumaric acid esters have been reported [2–4]. In one case no severe lymphopenia was detected as the reported nadir of lymphocytic count was 792/μl [5]. The active antipsoriatic agent monomethylfumarate inhibits the expression of proinflammatory cytokines, tumor necrosis factor alpha, interleukin-6, interleukin-1 alpha [6] and increases the secretion of interleukin-4 and interleukin-10 by helper T-cells [7]. Dimethyl fumarate was also introduced as an oral drug for relapsing remitting forms of multiples sclerosis (MS) [8]. Regarding the fact that we are already facing the problem of inducing PML in MS patients treated with natalizumab and dimethyl fumarate becoming an alternative drug in these cases, it is of major interest to understand more about the risk of developing PML by fumarate therapy.

## Case presentation

**Fig. 1:**
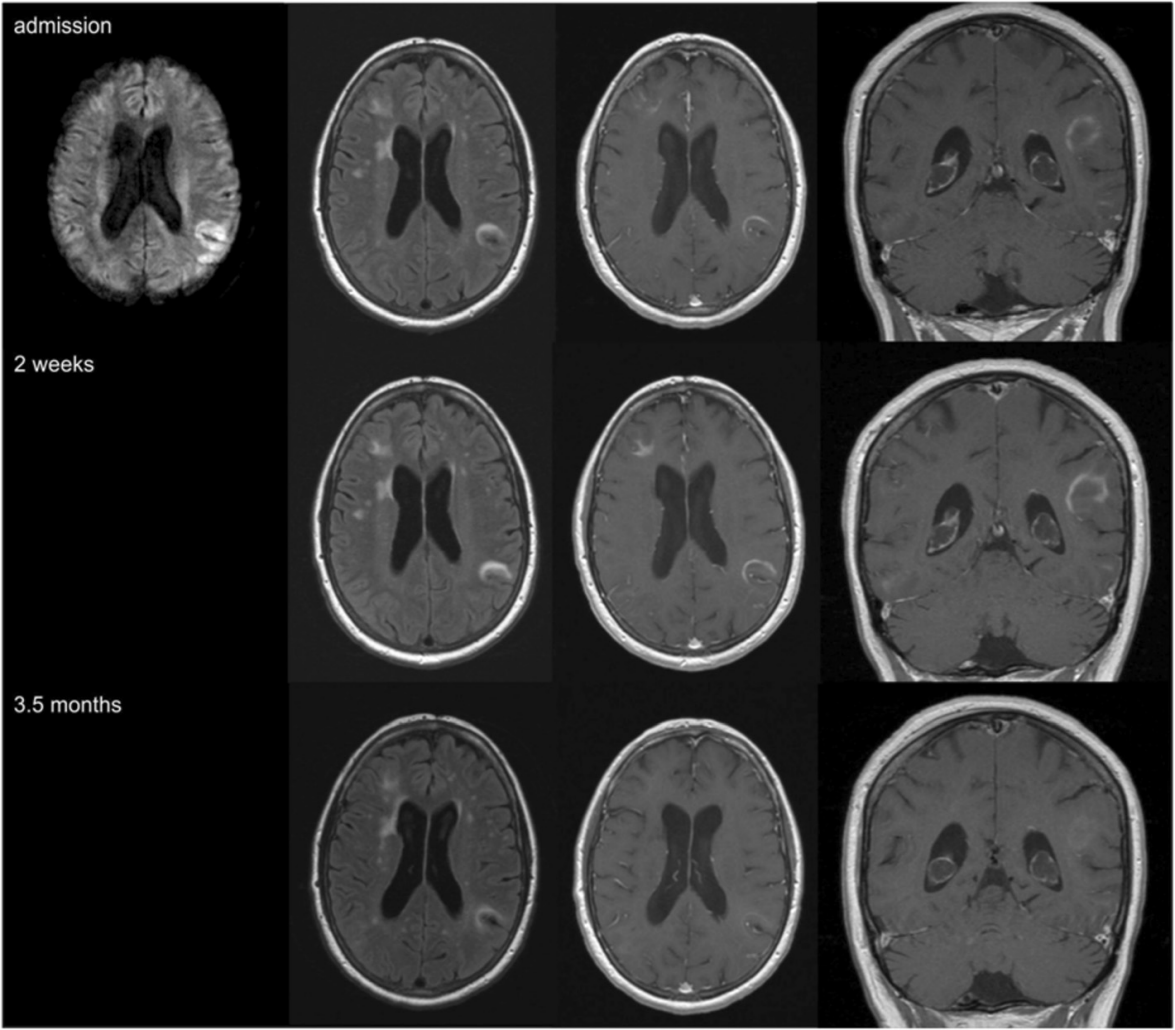
MRI of the patient’s brain in the course of the disease. shows MR imaging of the brain at the onset of symptoms and imaging controls after two weeks and 3.5 months. Several subcortical lesions are located in the right frontal and left parietal lobe and show progression during the first two weeks. They present hyperintense lesions in T2/FLAIR with partial increased signal in contrast-enhanced imaging.

A 53-year-old Caucasian woman reported to our clinic in September 2014 with a first and acute episode of transient confusion and sensory aphasia and a two days history of headache. She was on medication for arterial hypertension and hypothyroidism. Since May 2013 she received dimethyl fumarate (Fumaderm®) for the treatment of psoriasis. The patient only received topical treatment prior to dimethyl fumarate. Her clinical examination on admission was unremarkable. Cerebral magnet resonance imaging (MRI) showed several subcortical T2 hyperintense and partially contrast-enhancing lesions in the right frontal and left parietal lobes, which were increased in size on follow-up MRI two weeks later (Fig. 1).

**Fig. 2:**
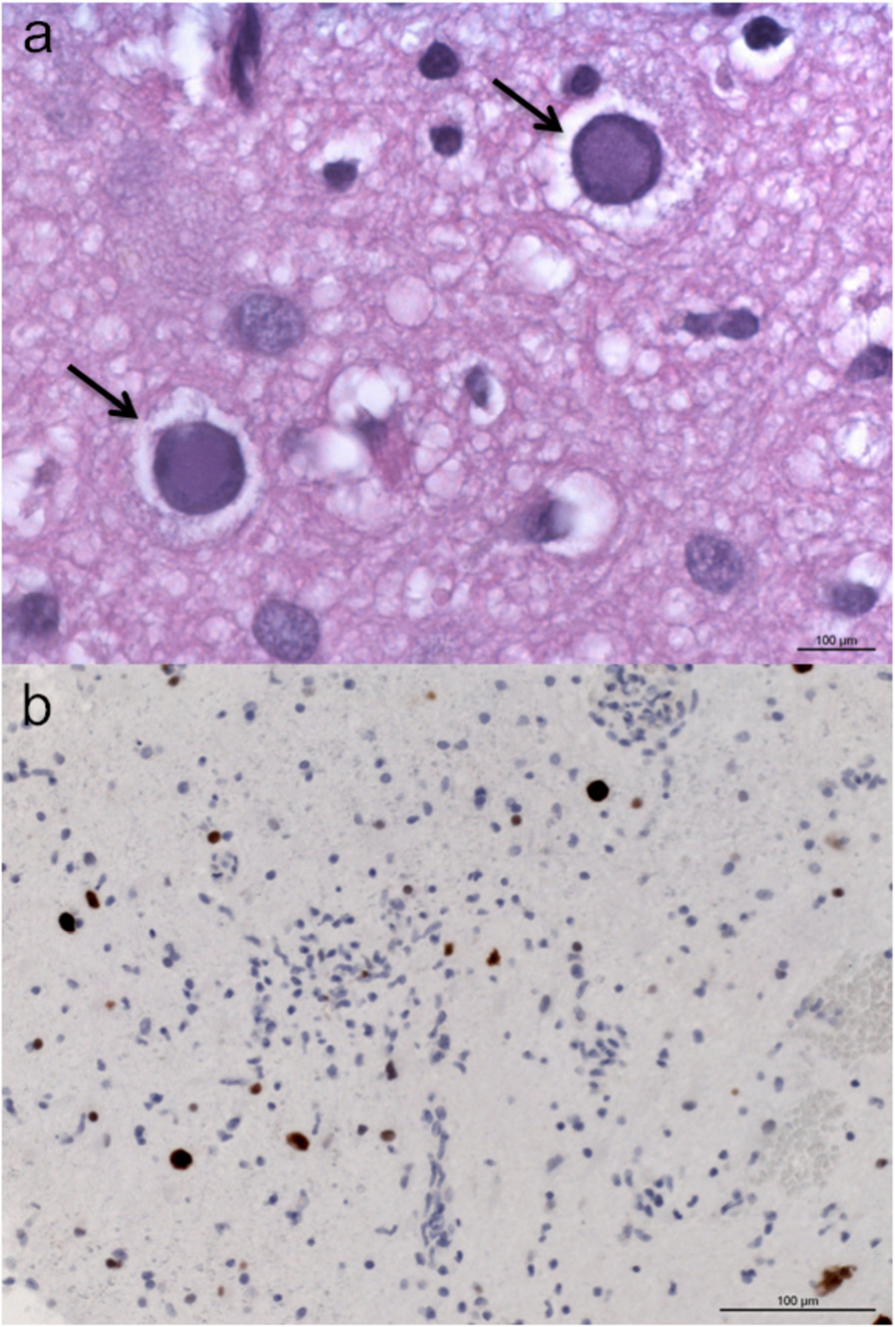
Histological analysis of a brain biopsy from the right frontal lesion. (a) Enlarged nuclei containing viral inclusions (arrows). Hematoxylin and eosin. (b) Enlarged nuclei appear immunopositive when labeled with an antibody to JC-virus/SV40.

Cerebrospinal fluid (CSF) protein was slightly elevated (54 mg/l), cell count and IgG-Index were normal and she had identical oligoclonal bands in serum and CSF. She did not have antibodies against (Human immunodeficiency virus) HIV 1 and 2 and polymerase chain reaction (PCR) for JC-virus desoxyribonucleic acid (DNA) (University Tübingen (sensitivity of JC-virus DNA: > 1000 copies) and University Düsseldorf (sensitivity of JC-virus DNA: > 4 copies/assay [9]), Germany) of CSF was negative. Histological analysis of a biopsy of the right frontal lesion showed infiltration of lymphocytes, perivascular CD45-positive cells, CD68-positive microglia, reactive astrocytosis and focal demyelination (Fig. 2). Some cells had intranuclear inclusions and stained positive with an antibody against JC virus. PCR of brain tissue for JCV DNA was positive and confirmed the diagnosis of PML.

**Fig. 3:**
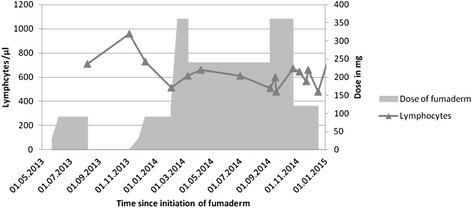
Absolute numbers of lymphocytes and dose of fumaric acid. After two months of application the lymphocyte count fell to a level of 700 cells/μl. At that point treatment was stopped until lymphocyte count recovered to a number of 900/μl after two and a half months. Dimethyl fumarate was given in an increasing dose up to 360 mg per day. When the symptoms of the psoriasis remained stable, the daily dose was decreased to 240 mg and increased again when the symptoms remitted. At the same time lymphocytes remained at an average level of about 600/μl. When they first fell under 500 cells/μl in October 2014, dimethyl fumarate dose was reduced to 120 mg. Finally, the therapy was stopped by the diagnosis of a PML. During the following months lymphocytes recovered slowly and reaching a number of 3000 – 5000 cells/μl.

Treatment with dimethyl fumarate was stopped and she was put on 250 mg Mefloquin per week and 45 mg Mirtazapin per day. The patients peripheral lymphocyte counts during treatment with dimethyl fumarate were retrospectively evaluated (Fig. 3) and ranged between 450 and 700 cells/μl while her leucocyte counts were higher than 3000/μl during the whole treatment period. The initial symptoms were considered to be epileptic and treatment with 1000 mg/day Levetiracetam was initiated. Upon follow-up three months later, the patient reported of persistent difficulties with concentration and word retrieval. Aphasia could not be detected in simple conversation and bed side tests. Neurological examination as well as repeated cerebral MRI showed no evidence for disease progression or immune reconstitution inflammatory syndrome. Upon 7-month follow-up the patient reported a stable course of disease with unchanged minor difficulties with concentration and word retrieval, which were very mild and could not be detected in conversation or neurological examination. The MRI showed an increase of the T2 hyperintense lesion in the right frontal lobe, while gadolinium enhancement and the other lesions remained unaltered. The patient tolerated the treatment well and reported no adverse events.

## Conclusions

A few cases of PML in patients treated with fumarates have been reported [3, 10, 11]. This case demonstrates that clinical examination, imaging of the brain and a sensitive JCV DNA PCR of CSF may not be sufficient to establish the diagnosis. There is evidence that an important risk factor for the development of PML is prolonged therapy-associated lymphopenia [12]. In these cases, discontinuation of the treatment is inevitable. The recommended acceptable threshold for therapy-associated lymphopenia is specific for any given drug. While the dosage of dimethyl fumarate has to be reduced when lymphocyte counts are lower than 700/μl, this threshold is 200/μl for fingolimod. Our patient demonstrates that PML can occur in patients with lymphocyte counts ranging between 450 and 700/μl for prolonged time periods. In another reported case, the lymphocyte nadir was 792/μl, indicating that no severe lymphopenia is required for PML to develop [5]. An alteration of immune surveillance for months or years appears to be critical for the development PML as it is the case in patients treated with natalizumab or efalizumab [13]. This case further demonstrates that PML may cause no or only faint symptoms and is not excluded by negative sensitive PCR for JCV DNA. Sensitivity of the assay used in the reference laboratory in Düsseldorf was 3.9 copies/assay (117 copies/ml), as they used an amplification protocol identical to the one used by Ryschekowitsch et al. [9]. As the sensitivity in various laboratories differs it could be necessary to analyse the CSF in a more sensitive laboratory in case of a negative result. Moreover, reactivation of JCV may not only lead to PML, but also to JCV associated granule cell neuronopathy [4, 14, 15], JCV encephalopathy [16] or JCV associated meningitis [17].

The incidence of PML in patients with long term immunomodulating or immunosuppressive therapy is thus possibly currently underestimated and a more careful surveillance of these patients would be necessary.

## Consent

Written informed consent was obtained from the patient for publication of this Case report and any accompanying images. A copy of the written consent is available for review by the Editor of this journal.

## Abbreviation

CNS: central nervous system
CSF: cerebrospinal fluid
DNA: desoxyribonucleic acid
HIV-1/HIV-2: human immunodeficiency virus
JC-Virus: polyoma JC-virus
MRI: magnet resonance imaging
MS: multiples sclerosis
PCR: polymerase chain reaction
PML: progressive multifocal leukoencephalopathy
